# Normal Leptin Expression, Lower Adipogenic Ability, Decreased Leptin Receptor and Hyposensitivity to Leptin in Adolescent Idiopathic Scoliosis

**DOI:** 10.1371/journal.pone.0036648

**Published:** 2012-05-15

**Authors:** Guoyan Liang, Wenjie Gao, Anjing Liang, Wei Ye, Yan Peng, Liangming Zhang, Swarkar Sharma, Peiqiang Su, Dongsheng Huang

**Affiliations:** 1 Department of Orthopedics, Sun Yat-sen Memorial Hospital of Sun Yat-sen University, Guangzhou, Guangdong, China; 2 Seay Center for Musculoskeletal Research, Texas Scottish Rite Hospital for Children, Dallas, Texas, United States of America; 3 Department of Spine Surgery, The First Affiliated Hospital of Sun Yat-sen University, Guangzhou, Guangdong, China; University of Tampere, Finland

## Abstract

Leptin has been suggested to play a role in the etiology of Adolescent Idiopathic Scoliosis (AIS), however, the leptin levels in AIS girls are still a discrepancy, and no *in vitro* study of leptin in AIS is reported. We took a series of case-control studies, trying to understand whether *Leptin* gene polymorphisms are involved in the etiology of the AIS or the change in leptin level is a secondary event, to assess the level of leptin receptor, and to evaluate the differences of response to leptin between AIS cases and controls. We screened all exons of *Leptin* gene in 45 cases and 45 controls and selected six tag SNPs to cover all the observed variations. Association analysis in 446 AIS patients and 550 healthy controls showed no association between the polymorphisms of *Leptin* gene and susceptibility/severity to AIS. Moreover, adipogenesis assay of bone mesenchymal stem cells (MSCs) suggested that the adipogenic ability of MSCs from AIS girls was lower than controls. After adjusting the differentiation rate, expressions of leptin and leptin receptor were similar between two groups. Meanwhile, osteogenesis assay of MSC showed the leptin level was similar after adjusting the differentiation rate, but the leptin receptor level was decreased in induced AIS osteoblasts. Immunocytochemistry and western blot analysis showed less leptin receptors expressed in AIS group. Furthermore, factorial designed studies with adipogenesis and osteogenesis revealed that the MSCs from patients have no response to leptin treatment. Our results suggested that *Leptin* gene variations are not associated with AIS and low serum leptin probably is a secondary outcome which may be related to the low capability of adipogenesis in AIS. The decreased leptin receptor levels may lead to the hyposensitivity to leptin. These findings implied that abnormal peripheral leptin signaling plays an important role in the pathological mechanism of AIS.

## Introduction

Adolescent idiopathic scoliosis (AIS) is a common tridimensional deformity, characterized by rotation of the vertebrae and lateral deviation of the spine. So far, the exact etiology of AIS remains elusive. It is generally accepted that AIS is a systemic disease and the scoliosis mainly results from the abnormal systemic skeletal growth and the asynchronous spinal neuro-osseous growth [1], [2], [3]. Also, AIS has been observed as a complex genetic disorder, and recent genome-wide association studies have implicated some new candidate genes [1], [2], [4], [5], [6]. Interestingly, several studies had found the AIS patients (especially in girls) have common features of taller stature, lower body mass index (BMI) and systemic low bone mass [7], [8], [9], [10], [11], which may be owing to a cytokine-like protein hormone: leptin [3], [11].

Leptin is coded by the *Leptin* gene (i.e. the obese gene, Ob) and is primarily expressed in white adipose tissue. It binds to leptin receptors and plays key roles not only in regulating the energy intake and expenditure of the body, but also in connecting the changes in body composition with bone formation and resorption [12], [13], [14]. Leptin affects bone metabolism via central and peripheral ways. It modulates cortical bone formation by regulating the expression of several neuropeptides in hypothalamus and inducing sympathetic activation [12], [15], [16]. It also directs the bone marrow stromal cells to osteogenic instead of adipogenic pathway [17], [18]. Thus an abnormal leptin level or the deficiency of signal pathway may result as a disorder in skeletal growth.

Leptin and its signaling pathway may be a candidate for the etiology of AIS. Significantly lower serum leptin levels were found in girls with AIS, and the leptin levels also correlated significantly with body weight, BMI and body mineral density (BMD) [19]. However, recently the same group claimed that the serum total leptin level between AIS and healthy girls are similar after adjusting the BMI [20]. Both of the studies were conducted with blood samples of patients, but *in vitro* experiment has not been reported. So we believe more input is needed for the leptin expression in AIS, and cytological evidences are warranted to gain deep insights.

Leptin being a very plausible candidate in AIS, it might be a very interesting question to begin with whether the alteration of leptin level is a primary event (i.e. as a result of variations in the gene) or secondary one (i.e. as an outcome). Association study of the polymorphisms in *Leptin* gene promoter did not find significant differences between cases and controls [21]. However, studies of polymorphisms in exons and untranslated regions of *Leptin* gene, which may as well influence the synthesizing and splicing of leptin, are lacking. The secretion of leptin is regulated secondary by other factors. Melatonin, which was widely considered acting a potential role in the onset and progression of AIS, has several effects in obesity-related metabolic alterations [22]. The defect of melatonin may lead to the change of leptin level, resulting in disorder of the leptin-hypothalamic-sympathetic nervous system, and bringing about the disease [23].

The adipogenic ability may affect the leptin level as well. The adipogenic ability impacts the number and size of adipocytes, and has great influence on the somatotype [24], [25]. There had been proposed an intrinsic relationship between the adipogenic ability and BMI [26]. AIS patients had been thought to have ectomorphic component with lower BMI, characterizing with less adipose tissue, and expressing less leptin [19]. Thus the adipogenic ability in AIS may have a relationship with the expression level of leptin. To evaluate the adipogenic ability, mesenchymal stem cells (MSCs) as the precursor cells of adipocytes are recommended for *in vitro* study [27], [28]. Although normal adipogenic ability of MSCs in AIS patients was reported, the researcher used Oil Red O staining and quantified with optical densities, which is lack of calibrator and is inaccurate due to the confounding factor of various cell numbers [29]. So, more insight in adipogenic capability of MSCs from AIS is needed.

Additionally, errors in function of leptin have been speculated in AIS patients [23]. Low serum leptin level stimulates fat accumulation through central and peripheral pathways and keeps the body in normal size, but in AIS patients this negative feedback seems to become invalid. Few studies were conducted to evaluate the role of leptin receptor. More soluble leptin receptor (sOB-R) was found in blood samples from AIS girls, and the abnormal leptin bioavailability was thought to play an important role in the initiation and progression of AIS [20]. However, the response to leptin in AIS was not reported. Hence, more details about leptin receptor and the peripheral response are needed.

Taking all this in consideration and to answer these questions, we designed a series of genetic and cytological studies. Firstly, we conducted the detection of single-nucleotide polymorphisms (SNPs) present in the *Leptin* gene and did a case-control association study, in order to examine whether the *Leptin* gene variations are associated with genetic susceptibility or curve severity in AIS. Secondly, we performed adipogenesis assays with bone MSCs, to evaluate the ability of adipogenesis and to detect the expression of leptin and leptin receptor in AIS adipocytes. Thirdly, we performed osteogenesis assays to evaluate the expression of leptin and leptin receptor in AIS osteoblast. Finally, we performed factorial designed studies, to examine the responses to leptin treatment during the adipogenic and osteogenic differentiation of MSCs. Our result demonstrated that: (1) the polymorphisms of *Leptin* gene have no association with genetic susceptibility/severity of AIS, and the leptin gene expression was normal in AIS induced adipocytes and osteoblasts after adjustment of the differentiation rate, indicating the change of serum leptin level probably is a secondary event; (2) low capability of adipogenesis is found in AIS girls; (3) the expression of leptin receptor is decreased in AIS which may lead to (4) hyposensitivity of AIS to leptin. The abnormal response to leptin may have a role in the pathogenesis of AIS.

**Figure 1:**
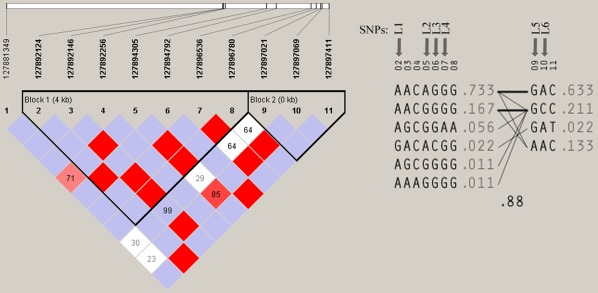
Linkage disequilibrium (LD) pattern and Haplotypes of the *Leptin* gene from the 45 healthy subjects. Two LD blocks were identified in the sequenced genomic region of the gene (calculated with the Solid spine of LD algorithm with the Minor Allele Frequency≥1%). Arrows indicate the positions of the 6 tag SNPs selected for the association study.

**Table 1:** 6 tag SNPs selected for the association study.

SNPs	Genome position(chr 7)	Reference SNP ID in NCBI	Location	Nucleotide substitutions	Residue change
L1	127892124	–	Coding region	c.53A>G	p.Tyr18Cys
L2	127894305	rs3828942	Intron	c.145–152G>A	–
L3	127894792	rs75506045	Coding region	c.480G>C	p.Gln160His
L4	127896536	rs10954174	3'-UTR	c.*1720A>G	–
L5	127897021	rs41457646	3'-UTR	c.*2205G>A	–
L6	127897069	rs11761556	3'-UTR	c.*2253A>C	–

## Results

### Genetic Association Study

We performed genetic association study in a systemic fashion. Screening of exons of the *Leptin* gene in 45 cases and 45 controls resulted in identification of 19 variations (data not shown). We did not see obvious accumulation of deleterious rare variations in cases as compared to controls. We further selected 11 of these variations with minor allele frequency (MAF) ≥1% from 45 sequenced healthy subjects to identify tag SNPs, and conducted an association study in 446 AIS patients and 550 healthy controls.

#### LD structure and tagging SNPs

Two LD blocks were revealed by default settings of haploview, and the frequencies of the haplotypes in each of them were observed. We identified 6 tag SNPs named L1 to L6 as shown in Figure 1 and Table 1.

#### Case-control association study

Age, gender and BMI are significantly different between the two groups, and therefore are in need of adjustment (Table 2). The genotype distributions of the 6 SNPs, which were shown in Table 3, were all in HWE. There were no statistically significant differences of any SNPs between the patients and controls. The results indicated no obvious association between any of the tag SNPs and AIS.

**Table 2:** Summary of the AIS and control subjects in genetic association study.

	AIS	Control	*P* value
Age (years)	10y –19y(average = 15.74y)	10y –21y(average = 14.02y)	0.001**
Gender	61 Male vs. 385 Female	48 Male vs. 502 Female	0.013*
Height (cm)	158.50±4.82	152.29±6.01	0.001**
Weight (kg)	42.76±4.01	43.07±7.09	0.57
BMI (kg/m^2^)	17.03±1.47	18.51±2.54	0.009**
cBMI (kg/m^2^)	16.79±1.47	18.51±2.54	0.001**

* *P*<0.05 was considered statistically significant.

** *P*<0.01.

**Table 3:** Results of the Case-control association studies.

SNPs	Genotype^a^		P-value		OR (95%CI)^c^
	Case(n = 446)	Control(n = 550)	Allele	Genotype^b^	
L1	441/5/0	545/5/0	0.739	0.922	1.234(0.356–4.278)
L2	127/228/91	166/291/93	0.246	0.885	1.111(0.930–1.327)
L3	444/2/0	548/2/0	0.834	0.999	0.811(0.114–5.766)
L4	298/148/0	396/148/6	0.209	0.965	1.169(0.916–1.491)
L5	354/88/4	452/96/2	0.213	0.648	1.206(0.898–1.620)
L6	225/188/33	290/222/38	0.492	0.944	1.071(0.880–1.305)

a The three values in the “genotype” column indicate the numbers of homozygotes(major allele)/heterozygotes/homozygotes(minor allele) in each SNP, respectively.

b After adjusting for age, gender and BMI by logistic regression.

c Calculated for the alleles.

Bonferroni adjustment (All SNPs *P*_adjusted_ = 1).

#### Genotype-phenotype studies

We further evaluated if there is any association of phenotypes with genotypes (Table 4). Although significant *P* value (at *P*<0.05) were observed for the maximum Cobb angles (MCAs) and different genotypes of rs11761556 (L6), it appears to be a false positive association as the average MCAs between three genotypes were almost similar.

As such, no association was observed between the *Leptin* gene variations and susceptibility/progression of the disease, suggesting that the low leptin level is a secondary outcome. Thus we tried to explore more functional details through cytology experiments, to figure out whether there is any factor responsible for the low serum leptin level in AIS patients.

**Table 4:** Average of the maximum Cobb angles in different genotypes.

SNPs	Average of MCA (Maximum Cobb Angle)^a^	*P* value of Kruskal-Wallis Test
L1	27.80±16°/38.00±16°/−c	0.108
L2	26.77±15°/29.37±19°/25.89±15°	0.462
L3	27.96±17°/19.00±9°/−	0.412
L4	27.74±16°/28.28±19°/−	0.996
L5	28.00±17°/28.00±16°/18.75±5°	0.598
L6	25.12±18°/25.76±16°/29.80±18°	0.012^b^

a The three values in the “Average of MCA” column indicated the mean MCA±Standard Deviation of homozygotes (major allele)/heterozygotes/homozygotes (minor allele) in each SNP, respectively.

b *P*<0.05 was considered statistically significant.

c No sample with homozygotes of minor alleles had been detected in L1, L3 and L4.

### Cytological Experiments

#### Summary of all subjects

Table 5 lists a summary of the demographic information of MSCs donors. The gender distribution between two groups was unmatched and the results need to be discussed based on gender. Although the average ages were similar in two groups, age as the potential confounding effects should be taken into account. So we compared the two groups with adjustment of age. The abnormality of anthropometric parameters in AIS patients had been widely described in previous researches, thus the significant differences in weight, BMI and cBMI between two groups might not be considered as sampling errors [7], [9], [11]. Given the intrinsic relationship between BMI and the capacity of adipogenesis, we did not conduct rectifications with BMI [26].

**Table 5:** Summary of the AIS and control subjects in cytological study.

	AIS	Control	*P* value
Age (years)	10y –20y(average = 15.21y)	10y –31y(average = 17.43y)	0.107
Gender	14 Male vs. 41 Female	18 Male vs. 10 Female	0.001**
Height (cm)	155.86±8.54	161.25±7.65	0.221
Weight (kg)	41.99±7.07	49.83±9.85	0.038*
BMI (kg/m^2^)	17.19±1.71	19.11±2.93	0.036*
cBMI (kg/m^2^)	16.33±1.86	19.11±2.93	0.006**

* *P*<0.05 was considered statistically significant.

** *P*<0.01.

#### Identification of MSCs

MSCs were isolated and purified as previously described. These MSCs were verified morphologically by a fibroblast-like appearance, and phenotypically by expression of CD29, CD44, CD105 but not CD34 and CD45 (data not shown).

### Comparing the Adipogenic Ability

To assess the expression of leptin in adipocytes and compare the adipogenic ability of MSCs between two groups, adipogenesis assay was performed for 12 days. The amount of lipids in induced adipocytes from AIS group, revealed by Oil Red O staining, was less than that from control group. We also quantitively investigated the expressions of Leptin, Leptin-R, SOCS3 and other adipogenic markers including PPARγ2, LPL and APN.

**Figure 2:**
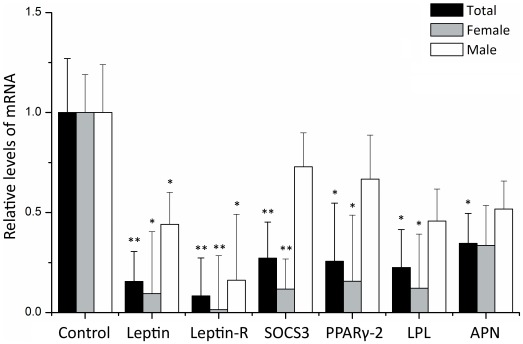
Relative levels of the genes in induced adipocytes from AIS group versus control group. The expression of target genes was measured by real-time quantitative RT-PCR and normalized to GAPDH expression. Relative expression levels were calculated by using the 2^−ΔΔCt^ method. The total group included the whole data set whereas the female and male groups included the data from each gender only. *P<0.05 was considered statistically significant. **P<0.01. All *P* values were calculated with age adjustment.

We compared the genes between the AIS and the control groups. All of the genes were observed with reduced expression in AIS group, suggesting the adipogenic ability of MSCs in AIS patient is lower (see Figure 2 and Table 6). The situations were a little different when compared the case-control sets based on their gender. In the female subjects, all genes were reduced significantly in AIS patients except APN. In the male subjects, however, only the expressions of Leptin and Leptin-R decreased significantly. Nevertheless, when used PPARγ2 as a reference gene to adjust the adipogenic differentiation rate, we found that the Leptin and Leptin-R gene in AIS group decreased insignificantly in both genders (*P*>0.05).

**Table 6:** Comparison of all genes between AIS and control groups in adipogenesis assay.

	Leptin	Leptin-R	SOCS3	PPARγ2	LPL	APN
Total	0.007**	<0.001**	0.001**	0.019*	0.019*	0.030*
Female	0.043*	0.009**	0.001**	0.041*	0.021*	0.090
Male	0.040*	0.032*	0.160	0.408	0.463	0.548

All *P* values were calculated with age adjusted.

* *P*<0.05 was considered statistically significant.

** *P*<0.01.

#### Relationship between the genes and clinical parameters

Correlations between the ΔCt value of all genes and the clinical parameters in both genders of AIS patients were summarized in Table 7.The levels of adipogenic markers (i.e. PPARγ2, LPL and APN) and Leptin showed good consistencies with each other. Nevertheless, the levels of Leptin-R and SOCS3 generally have no correlation with the adipogenic markers (except Leptin-R vs. LPL). The Cobb angle did not correlate with the level of all genes and BMI, suggesting the low adipogenic ability may not directly impact the severity of the disease.

**Table 7:** r values of Correlations between the genes and parameters in AIS patients.

	PPARγ2	LPL	APN	Leptin	Leptin-R	SOCS3	Cobb	BMI	cBMI
PPARγ2	–	0.514**	0.578**	0.292*	0.098	−0.075	−0.063	−0.076	−0.033
LPL	0.514**	–	0.569**	0.331*	0.339*	0.012	−0.070	−0.132	−0.060
APN	0.578**	0.569**	–	0.426**	0.287	−0.025	−0.166	−0.259	−0.197
Leptin	0.292*	0.331*	0.426**	–	0.697**	0.275	−0.033	−0.115	−0.084
Leptin-R	0.098	0.339*	0.287	0.697**	–	0.167	−0.120	−0.120	−0.043
SOCS3	−0.075	0.012	−0.025	0.275	0.167	–	−0.077	−0.204	−0.248
Cobb	−0.063	−0.070	−0.166	−0.033	−0.120	−0.077	–	−0.002	−0.321*
BMI	−0.076	−0.132	−0.259	−0.115	−0.120	−0.204	−0.002	–	0.878**
cBMI	−0.033	−0.060	−0.197	−0.084	−0.043	−0.248	−0.321*	0.878**	–

* *P*<0.05 was considered statistically significant.

** *P*<0.01.

#### Leptin receptor expression in induced osteoblast

**Figure 3:**
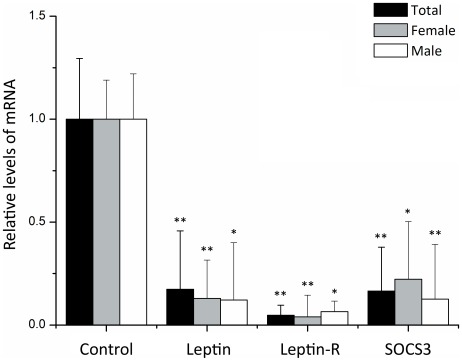
Relative levels of the genes in induced osteoblasts from AIS group versus control group. The expression of target genes was measured by real-time quantitative RT-PCR and normalized to GAPDH expression. Relative expression levels were calculated by using the 2^−ΔΔCt^ method. *P<0.05 was considered statistically significant. **P<0.01. All *P* values were calculated with age adjustment.

As shown in Figure 3, the expression of Leptin, Leptin-R and SOCS3 were significantly decreased in all AIS patients (*P*<0.001). The differences remain significant when compared the case-control sets based on their gender. When we used Runx2 gene as reference to adjust the different osteogenic rates between two groups, we found the Leptin-R in AIS still decreased significantly in total and female data set (*P* = 0.041 and *P* = 0.028, respectively), but in male subjects were similar (*P* = 0.239). The decrease of Leptin expression was not statistically significant after the Runx2 adjustment in both genders (*P*>0.05). No correlation was found between the level of any gene and Cobb angle (*P*>0.05).

#### Immunocytochemistry and western blot of Leptin receptor

**Figure 4:**
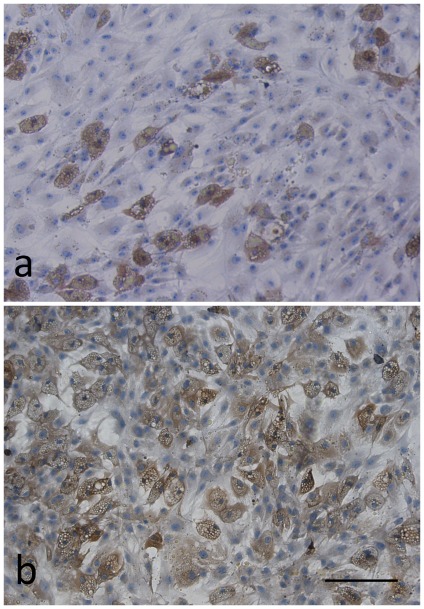
The expression of leptin receptor in induced adipocytes was determined by immunocytochemistry. The AIS MSCs (a) was from a 14-year-old boy (Cobb angle = 80°) and the normal control (b) was from a 19-year-old boy. Note that the leptin receptors were abundant in undifferentiated MSCs from control sample, but were rare in those from AIS sample. Scale bar = 200 µm.

**Figure 5:**
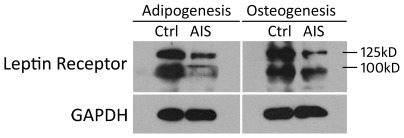
Western blot was performed to detect Leptin receptors in adipogenic and osteogenic MSCs. Two variants of Leptin receptors were present at ∼100 kD and ∼125 kD. The normal control sample (Ctrl) was from a 20-year-old girl and the AIS sample (AIS) was from a 15-year-old girl (Cobb angel = 68°). The level of leptin receptor was obviously lower in AIS sample.

For a single adipocyte, the Leptin receptors seemed to express at a similar level between AIS and control groups (Figure 4). However, the leptin receptors were abundant in undifferentiated MSCs from control sample, but were rare in those from AIS sample. Besides, the expression of Leptin receptor was found decreased by immunoblot in AIS induced adipocytes and osteoblasts (Figure 5).

### Responses to Leptin Treatment between AIS and Control MSCs

The osteogenesis assay implied that the leptin receptor may be involved in the progression of AIS. On the other hand, the low serum leptin in AIS can also be explained as the excessive sensitivity of the receptor or the signaling pathway, which may in turn reduce the leptin/leptin receptor levels through a negative feedback loop. So the effect of leptin in AIS patients was needed to be studied. Leptin had been reported to restrain adipogenesis and enhance osteogenesis [18]. We conducted factorial designed studies to examine the difference of responses to leptin treatment between AIS and control MSCs.

#### Effects of leptin in adipogenic differentiation

**Figure 6:**
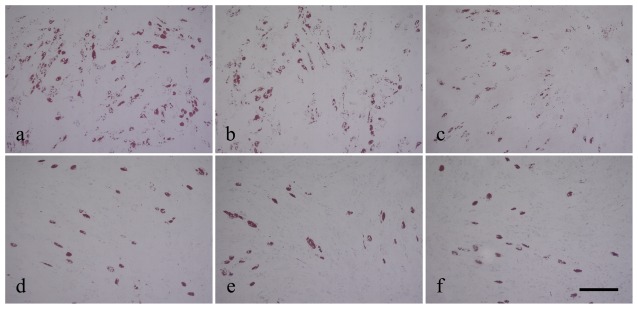
The effect of leptin on adipogenesis of MSCs from a normal control (18y, male) and an AIS patient (15y, male, Cobb angle = 54°). Cells from the normal control were induced along the adipogenic differentiation with human recombined leptin protein at concentration of 0 µg/mL (a), 0.015 µg/mL (b) and 0.6 µg/mL (c). MSCs from the AIS patient were also treated under the same condition that containing leptin of 0 µg/mL (d), 0.015 µg/mL (e) and 0.6 µg/mL (f). At day 12, the cells were subjected to Oil Red O staining. Scale bar = 500 µm.

Oil Red O staining (Figure 6) showed that the leptin treatment produced a dose-dependent reduction in the ability of adipogenesis of MSCs in control group. Specifically, the size and numbers of adipocytes were significantly decreased after treated with leptin at doses of 0.6 µg/mL. In contrast, leptin treatment brought no change in AIS group, and all of them displayed adipogenesis levels lower than in control group.

**Figure 7:**
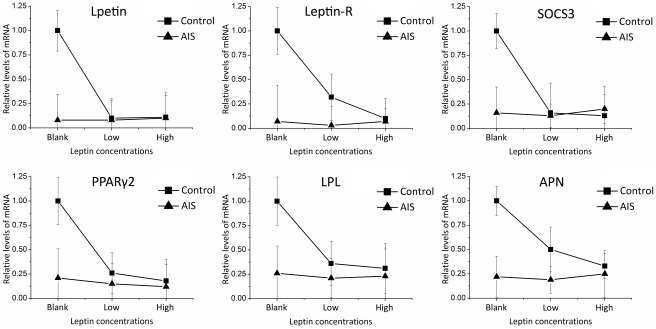
Gene expression levels in response to different doses of leptin. The expression levels in control group without leptin treatment were used as calibrators. Relative expression levels were calculated by using the 2^−ΔΔCt^ method.

The relative levels of several genes showed different responsive patterns between two groups in adipogenic differentiation (Figure 7 and Table 8). Significant Leptin × Status interaction existed in the expression of Leptin, Leptin-R and SOCS3. ANOVA analysis showed that leptin treatment reduced Leptin, Leptin-R and SOCS3 expression significantly in control group, but had no influence on those in AIS group, eliminating the difference between two groups. Along with the increase of leptin concentration, the changes of PPARγ2, LPL and APN between the two groups had slight but nonsignificant differences. All the genes in AIS group sustained in similar levels, implying the induced adipocytes in AIS group are insensitive to leptin treatment.

**Table 8:** *P* values of ANOVA for factorial designed data in Adipogenesis assay.

		Leptin	Leptin-R	SOCS3	PPARγ2	LPL	APN
Leptin × Status interaction		0.031*	0.048*	0.043*	0.401	0.674	0.451
Main effect of Status		0.045*	0.132	0.123	0.018*	0.156	0.043*
Main effect of Leptin		0.016*	0.001**	0.093	0.034	0.532	0.569
Difference within different leptin concentration	Control group	0.027*	0.045*	0.027*	0.065	0.477	0.347
	AIS group	0.847	0.745	0.660	0.554	0.968	0.945
Difference between AIS and control	Blank	0.010*	0.004**	0.035*	0.011*	0.139	0.010*
	Low Leptin level	0.722	0.609	0.666	0.374	0.538	0.255
	High Leptin level	0.779	0.869	0.503	0.455	0.741	0.804

* *P*<0.05 was considered statistically significant.

** *P*<0.01.

#### Effects of leptin in osteogenic differentiation

**Figure 8:**
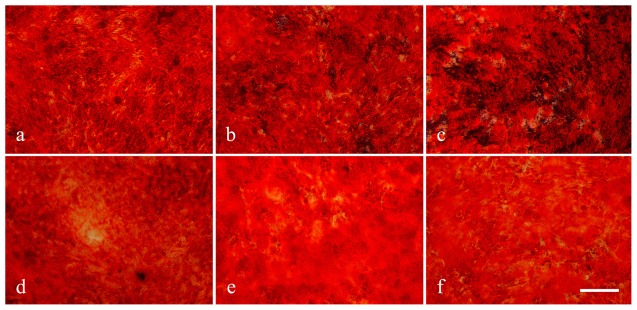
The effect of leptin on osteogenesis of MSCs from a normal control (22y, male) and an AIS patient (18y, male, Cobb angle = 88°). Cells from the normal control were induced along the osteogenic differentiation with human recombine leptin protein at concentration of 0 µg/mL (a), 0.015 µg/mL (b) and 0.6 µg/mL (c). MSCs from the AIS patient were also treated under the same condition that contained leptin of 0 µg/mL (d), 0.015 µg/mL (e) and 0.6 µg/mL (f). At day 21, the cells were subjected to Alizarin Red S staining. Scale bar = 500 µm.

Alizarin Red S staining (Figure 8) showed that leptin dramatically promoted mineralization of induced osteoblast in control group, but not in AIS group. The osteogenic ability of MSCs from AIS group was lower than control when cultured without and with different doses of leptin.

**Figure 9:**
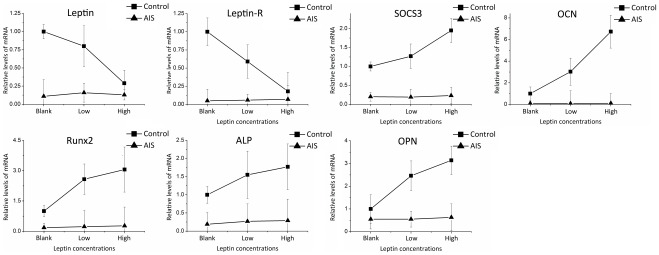
Gene expression levels in response to different doses of leptin. The expression levels in control group without leptin treatment were used as calibrators. Relative expression levels were calculated by using the 2^−ΔΔCt^ method.

We also investigated the effects of leptin on expression of several genes during osteogenesis (Figure 9 and Table 9). Significant Leptin × Status interaction were found in the expression of Leptin, Leptin-R, OCN and OPN. ANOVA results showed that leptin treatment reduced Leptin, Leptin-R and increased OCN, OPN significantly in control group. However, leptin did not have any effect on the expression of these genes in AIS group. Leptin also appears to promote the expression of SOCS3, Runx2 and ALP in control group, but statistically insignificant. All levels of SOCS3, OCN, Runx2 and ALP in AIS were significantly lower than control group, and the leptin treatment enlarged the differences, indicating a decreased reactivity to leptin in AIS induced osteoblast.

**Table 9:** *P* values of ANOVA for factorial designed data in Osteogenesis assay.

		Leptin	Leptin-R	SOCS3	OCN	Runx2	ALP	OPN
Leptin × Status interaction		0.048*	0.038*	0.525	0.034*	0.794	0.993	0.041*
Main effect of Status		<0.001**	<0.001**	<0.001**	<0.001**	<0.001**	<0.001**	0.004**
Main effect of Leptin		0.185	0.186	0.563	0.040	0.420	0.523	0.141
Difference withindifferent leptinconcentration	Control group	0.045*	0.040*	0.500	0.036*	0.587	0.762	0.045*
	AIS group	0.702	0.771	0.878	0.199	0.763	0.674	0.711
Difference betweenAIS and control	Blank	<0.001**	0.002**	0.022*	<0.001**	0.034*	0.020*	0.666
	Low Leptin level	0.042*	0.001**	0.001**	<0.001**	0.005**	0.008**	0.048*
	High Leptin level	0.016*	0.341	0.003**	<0.001**	0.014*	0.019*	0.002**

* *P*<0.05 was considered statistically significant.

** *P*<0.01.

## Discussion

This is the first *in vitro* study of leptin and leptin receptor in AIS patients. Our results indicated that *Leptin* gene variations are not associated with the susceptibility/progression of the disease, and the leptin gene expression was normal in AIS induced adipocytes and osteoblasts after adjustment of the differentiation rate, implying reduced leptin level is a secondary event. Moreover, in AIS the adipogenic capability of MSCs is lower, the leptin receptor level is decreased and the response to leptin is deficient.

Genetic association studies were expected to identify potential risk variants of AIS and recent GWAS identified some of the candidate genes [5], [6], [30]. However, such studies are warranted in Chinese population. This study confirmed that the genomic polymorphisms of *Leptin* gene are not associated with the genetic susceptibility to AIS, which are consistent with the finding of Morocz et al [21]. Despite a small *P* value was observed for the MCAs and different genotypes of L6, the average MCAs between the three genotypes were almost similar (25°/26°/30° with wide standard deviations), suggesting the observation is of no clinical significance.

Previously the adipogenesis levels in AIS group were considered similar to controls [29]. It was also reported that the osteogenic ability of MSCs from AIS patients is less, which correlates with their reduced bone mineral density. We repeated the osteogenesis assay in our factorial designed study (cultured without leptin) and found similar results. It has been considered that the adipocyte and osteoblast lineage pathways of MSCs have an inverse relationship [31], [32]. It is natural to deduce that MSCs from AIS may be prone to adipogenesis. Surprisingly, our investigations in adipogenic marker genes indicated that the MSCs from the AIS girls have a lower capability of adipogenesis. In order to remove any confounding effect of age distribution in our study, as MSCs from elder person may tend to undergo adipogenesis [33], we adjusted results for age and observed the adipogenic ability was still significantly lower in AIS group. Our results also showed that the leptin levels are similar between two groups after adjusting the adipogenic level, implying that the synthesis and secretion of leptin are normal in AIS, and the change of leptin level may be a secondary event. Our study not only explained the thin stature of AIS girls, but also implied that the low leptin level in AIS girls may have a relationship with lacking of fat cells. For confirmation, the relationship between serum leptin level and adipogenic ability needs further evaluation. Meanwhile, other factors which may affect the adipogenic ability, such as melatonin signaling, need investigation so as to figure out the primary event that causes the abnormal leptin level [34].

The hyposensitivity of leptin in MSCs from AIS group is elusive at the moment. We did not observed statistical significance in the Leptin × Status interaction of several marker genes, which may be mainly owing to the limitation of small sample size and great deviations within the subjects. However, the relative levels of the genes remarkably demonstrated the low response to leptin of AIS group. The rare leptin receptor in undifferentiated MSCs as well as the low level of Leptin-R in osteoblasts, are most likely to be the direct explanations of the hyposensitivity of leptin. Western blot also showed decreased leptin receptors in AIS induced adipocytes and osteoblasts, but these results may be confounded by the low adipogenesis and osteogenesis levels of AIS MSCs. Besides, the major leptin signaling pathway, namely JAK2/STAT3 pathway, may also have a role in the low response to leptin in AIS. The expression of SOCS3 is induced by the JAK2/STAT3 pathway and is reported to be an indicator of activity of the signaling [12], [35]. Leptin signaling is also subject to negative feedback regulation [12], and leptin treatment may reduce the expression of leptin and its receptor. Our results showed the expression of Leptin, Leptin-R and SOCS3 in AIS group were invariant no matter how much leptin exist. Thus we conclude that the defect of leptin signaling pathway, if present, may be located in JAK2/STAT3 pathway. Moreover, there can be another possibility. The abnormal MSCs are sensitive in none of adipogenesis, osteogenesis or leptin treatment, in other words, the AIS MSCs have lower activity. So the hyposensitivity of leptin is probably one of the behaviors of the inactivity of the MSCs from AIS.

The leptin signaling may contribute to the etiology of AIS. Leptin is one of the key regulators in pubertal growth and development. It had been shown to mediate the bone metabolism and remodeling in vitro and in vivo, in peripheral and in central nervous system [12], [13], [17], [36], [37]. In the leptin receptor-deficient db/db mouse, where leptin signaling is absent, bone mass and strength are reduced [38]. Leptin can rescue the skeletal length and bone mineral density in leptin-deficient ob/ob mouse [39], [40]. In AIS patients, an earlier study found the serum leptin level correlated with bone mineral density, and another study showed a relationship between free leptin index and curve magnitude in AIS [19], [20]. In this study, we found reduction of Leptin-R level and hyposensitivity of leptin in AIS. Although there is not enough evidence to prove the relationship between the hyposensitivity and the onset of AIS, bipedal db/db mouse model can be constructed and the role of leptin signaling can be confirmed.

There are some limitations that should be considered in this study. One of them is the restriction of the small sample size and the great deviation of subjects, and thus we could not examine the response to leptin between different genders. The result may be more convincing if replication is performed. Another weakness is that the relative level of marker genes may not completely reflect the differentiation level of MSCs. Oil Red O quantified with optical densities and alkaline phosphatase activity assay may be further performed to examine the response to leptin in an overall perspective.

In conclusion, our cytological experimental data emphasized the potential role of leptin signaling in the etiopathogenesis of AIS. Next step of the studies may be focused on whether and how the defect of peripheral leptin signaling acts on the body and induces skeletal deformities.

## Materials and Methods

This series of studies were approved by the ethics committee of Sun Yat-Sen University, and written informed consent was obtained from all individuals and/or their parents.

### Association Study

#### Subjects

The study consisted of 446 AIS patients recruited from the scoliosis clinic. Diagnoses of the patients were confirmed by experienced surgeons using the Adams forward bending test and posteroanterior radiographic images of the whole spine. The inclusion criteria are existing rotational prominence and a maximum Cobb angle above 10°. Patients with scoliosis secondary to congenital vertebral malformation, neuromuscular disorders or syndromic disorders were excluded from the study. Patient’s clinical information, including age, gender, curve pattern, Cobb angle, and Risser sign were recorded. Patients were followed till skeletal maturity (Risser sign grade 5) unless they accepted surgery. The minimum follow-up time is one year. If the patient was treated with bracing, the maximum value in the recorded Cobb angles was considered as Maximum Cobb Angle (MCA); otherwise, the Cobb angle obtained at the last visit was MCA. The maximum Cobb angle ranged from 10° to 140°.

Meanwhile, 550 healthy controls were recruited to the study. All the controls were examined with the forward bending test to exclude any scoliosis, and radiographs were taken for validation in case of any uncertainty. The controls were also eliminated if they had suffered from any congenital deformity of the spine or had a family history of scoliosis. All of the cases and controls were Han Chinese from south China. Informed consent to DNA analysis was signed by all subjects or their parents.

Blood samples were collected from each subject through venipuncture. Genomic DNA was isolated with Tiangen DNA blood Mini kits (Tiangen, Beijing, China) from peripheral blood samples collected from each subject.

#### Resequencing of the Leptin Gene and SNPs Identification

In order to screen variations in exons of the *Leptin* gene, at first resequencing was performed in 45 cases and 45 controls, which were randomly selected from the collection. Primers were designed for all exons and intron-exon boundaries of the gene, with 100–200 bp extensions into intronic regions (see Table S1). The PCR products were sequenced in both directions by ABI Sequence Analyzer 3730XL (Applied Biosystems, Foster City, CA, USA), and the results were then compared with sequences retrieved from the UCSC Genome Browser (http://genome.ucsc.edu/). We identified 19 variations, including 9 novel rare variations. Data of the novel variations has not been submitted to GenBank.

Linkage disequilibrium (LD) analysis, haplotype construction and tag SNP selection of the region were performed with Haploview 4.2 [41]. SNPs obtained from the 45 sequenced healthy subjects with minor allele frequency (MAF) ≥1% were selected for finding tag SNPs.

#### Genotyping methods

6 tag SNPs were identified for further analyses (see Table 1). They were then detected in 446 AIS patients and 360 healthy controls by the TaqMan-based genotyping assay. All of the primers and the probes are listed in the Table S2. The TaqMan-based genotyping assay was carried out with the ABI 7500 real-time PCR System (Applied Biosystems). The reaction mix contained ddH_2_O 3.25 µL, MgCl_2_ 3 µL, buffer 1 µL, dNTP 0.25 µL, Primers 0.2 µL, Probes 0.05 µL, ROX reference dye 0.05 µL and DNA template 2 µL, in a total volume of approximately 10 µL. Amplification was carried out with a 10 minute cycle at 95°C, 50 cycles at 95°C for 30 seconds and 63°C for 1 minute. The result was analyzed with the SDS v1.2×System Software (Applied Biosystems). For quality control in each plate, the sample genotypes confirmed by direct sequencing were used as positive controls and No Template Control as negative control.

### Cytological Experiments

Criterion for AIS subjects for cytological experiments were as follows: (1) diagnosis of AIS was physically and radiographically confirmed, (2) surgery at our institute, (3) no other skeletal deformity. Patients complicated with nutritional or metabolic diseases were excluded. Control subjects were recruited from patients with fractures. All of the control subjects were confirmed to have a straight spine by X-ray. Subjects with BMI>23 g/cm^2^ in both groups were also exclude to avoid overweight or obesity which may influence the adipogenic ability of MSCs. In total 55 AIS patients and 28 controls were recruited. Bone marrow samples were provided voluntarily and clinical information was record. For AIS patients, corrected height was calculated with Bjure’s formula (log *y* = 0.011*x* –0.177, where *y* stands for the loss of trunk height due to the deformity and *x* stands for maximum Cobb angle of the primary curve). Corrected BMI (cBMI) is determined by dividing weight (kg) by the square of the corrected height (m^2^).

#### Isolation and culture of MSCs

MSCs were isolated and purified by density gradient centrifugation combined with an attachment culture method [34]. Briefly, mononuclear cells in bone marrow were separated in a lymphoprep density gradient by centrifugation at 500 g for 20 minutes, suspended in Dulbecco’s modified Eagle’s medium (DMEM) with 10% fetal bovine serum (FBS), seeded and incubated at 37°C/5% CO_2_. Non-adherent cells were removed after 48 hr by changing the medium. Thereafter, the medium was changed every 3 days. When reached 80% confluences, cells were trypsinized (0.25% trypsin) and plated again. Cells from passages 3∼6 were used for the experiments.

#### Adipogenesis assay

For adipogenic induction, MSCs were plated at the density of 3×10^4^ cells/cm^2^ in 6-well plated. After grown to postconfluence, cells were cultured in an adipogenic medium for 12 days, which consisted of high-glucose DMEM, 10% FBS, 1 µM dexamethasone, 60 µM indomethacin, 500 µM 3-Isobutyl-1-methylxanthine, 0.01 mg/mL insulin.

#### Osteogenesis assay

It had been reported that the osteogenic ability of MSCs from AIS patients is lower than normal [29]. We conducted the osteogenic differentiation to evaluate the difference of expression of Leptin, Leptin-R and SOCS3 in osteoblast between two groups. Osteogenesis assay was performed on 20 AIS patients versus 17 normal controls (age and gender matched), which were selected randomly from our MSCs sample bank described above. Cells were plated and grown until confluence, then cultured with osteogenic medium for 12 days. The osteogenic medium contained low-glucose DMEM, 10%FBS, 0.1 µM dexamethasone, 10 mM β-glycerophosphate, and 50 µg/mL ascorbic acid.

#### Immunocytochemistry analysis

To examine expressions of Leptin receptor after adipogenesis, immunocytochemistry was performed with Hsitostain-Plus kit (ZSGB-BIO, Beijing, China). After fixed and permeabilized, cells were blocked and incubated with the antibodies against Leptin receptor (R&D systems, Minneapolis, MN, USA). Detection was conducted with a DAB Horseradish Peroxidase Color Development Kit (ZSGB-BIO, Beijing, China).

#### Western blot analysis

Total proteins were collected with RIPA buffer plus protease inhibitors from cells after adipogenic or osteogenic induction. Equal amounts (50 µg) of each sample were resolved on 10.5% SDS-PAGE gel and transferred to PVDF membranes. After blocked with 5% nonfat dry milk for 1h at room temperature, membranes were incubated with antibodies against Leptin receptor (R&D systems, Minneapolis, MN, USA) or GAPDH (R&D systems, Minneapolis, MN, USA). Antibody-specific labeling was revealed by incubation with a HRP-conjugated secondary antibody for 1 h and visualized with the ECL kit (Millipore, Billerica, MA, USA).

#### The difference of responses to leptin treatment between AIS and control MSCs

We also chose 19 AIS patients randomly and 19 age- and gender-matched normal controls for the factorial designed study. Cells were treated for 12 days in adipogenic medium or osteogenic medium without leptin, with low level leptin (0.015 µg/mL, the average circulating level of normal adolescent) [19] or high level leptin (0.6 µg/mL) [18], respectively. Intracellular lipid accumulation in the adipocyte was indicated with Oil Red O staining. The induced adipocytes was fixed in 4% paraformaldehyde for 1 hour after washed briefly, stained with newly filtered Oil Red O for 30 min at room temperature, then rinsed and photographed. Cells that had osteogenic differentiated for 21 days was indicated with Alizarin Red S staining and photographed.

#### Real-time RT-PCR assay

Total RNA was isolated from induced adipocyte or osteoblast with RNAiso Plus reagent (TaKaRa, Dalian, China) and converted to cDNA. Real-time PCR was performed on a Roche LightCycler 480 System using SYBR Green Realtime PCR Master Mix (TOYOBO, Osaka, Japan). The expression of the Leptin gene, Leptin receptor (Leptin-R, long form) gene and Suppressor of Cytokine Signaling 3 gene (SOCS3, which is induced by the JAK2/STAT3 pathway of leptin signaling and acts to inhibit the activation of leptin receptor [12]) were examined. Several adipogenic marker genes were also investigated, including peroxisome proliferator-activated receptor-gamma 2 (PPARγ2), lipoprotein lipase (LPL), adiponectin (APN). The genes of osteocalcin (OCN), Runt-related transcription factor 2 (Runx2), Alkaline phosphatase (ALP) and osteopontin (OPN) were chose to evaluate the osteogenic ability. Expression of GAPDH gene was used as reference. It’s noteworthy that the different differentiation rates between AIS and control in adipogenesis and osteogenesis may have undesired interferences when comparing the level of leptin and leptin receptor. So we need reference genes to adjust the differentiation rates. The PPARγ and Runx2 have a crucial role in adipogenesis and osteogenesis respectively and their level represent the differentiation level of the MSCs [28], [42]. So we use these genes as reference to calibrate the differentiation rates between AIS and control. The primer sequences employed are listed in the Table S3. Each reaction was processed in triplicate and average ΔCt value from the whole group was taken. Relative expression levels of each gene and the calibration of the differentiation rate were obtained by using the 2^−ΔΔCt^ method.

#### Statistical analyses

In the genetic association study, Hardy-Weinberg equilibrium (HWE) test was performed. The allelic association analyses were performed by Chi-square tests. Logistic regressions were used to adjust for the confounding effects of age and gender for genotypic association, while Bonferroni adjustment was performed for multiple-tests of all SNPs. In genotype-phenotype study, Kruskal-Wallis Test was used for the comparison of mean maximum Cobb angles among different genotypes in dataset from cases only, if the Cobb angles showed an abnormal distribution. In the studies of MSCs, t-test was performed to compare the ΔCt value of each gene between AIS and control group (with the null hypothesis: the Ct differences between target and reference genes will be the same in AIS vs. control samples), and the adjustment with age was performed with ANOVA. Test of Pearson’s correlation was used to evaluate the relationship within the genes, Maximum Cobb Angle, BMI and cBMI. The factorial designed study was analyzed with ANOVA for factorial designed data (univariate general linear model), where the Leptin × Status interaction was detected to indicate the differences of responses to leptin. All analyses were performed using the SPSS software (SPSS for Windows, Rel. 17.0.0. 2008. Chicago: SPSS Inc.).

## Supporting Information

Table S1 **All primers for resequencing in genetic association study.**
(DOC)

Table S2 **All primers and probes for each SNP in genetic association study.**
(DOC)

Table S3 **All primers for Real-time RT-PCR assay in cytological experiments.**
(DOC)

## References

[1] Wang WJ, Yeung HY, Chu WC, Tang NL, Lee KM (2011). Top theories for the etiopathogenesis of adolescent idiopathic scoliosis.. J Pediatr Orthop.

[2] Kouwenhoven JW, Castelein RM (2008). The pathogenesis of adolescent idiopathic scoliosis: review of the literature.. Spine (Phila Pa 1976).

[3] Lombardi G, Akoume MY, Colombini A, Moreau A, Banfi G (2011). Biochemistry of adolescent idiopathic scoliosis.. Adv Clin Chem.

[4] Cheung KM, Wang T, Qiu GX, Luk KD (2008). Recent advances in the aetiology of adolescent idiopathic scoliosis.. Int Orthop.

[5] Sharma S, Gao X, Londono D, Devroy SE, Mauldin KN (2011). Genome-wide association studies of adolescent idiopathic scoliosis suggest candidate susceptibility genes.. Hum Mol Genet.

[6] Takahashi Y, Kou I, Takahashi A, Johnson TA, Kono K (2011). A genome-wide association study identifies common variants near LBX1 associated with adolescent idiopathic scoliosis.. Nat Genet.

[7] Barrios C, Cortes S, Perez-Encinas C, Escriva MD, Benet I (2011). Anthropometry and Body Composition Profile of Girls with non Surgically-treated Adolescent Idiopathic Scoliosis.. Spine (Phila Pa.

[8] Sadat-Ali M, Al-Othman A, Bubshait D, Al-Dakheel D (2008). Does scoliosis causes low bone mass? A comparative study between siblings.. Eur Spine J.

[9] Siu KCC, Tak KLW, Kit TY, Ping TS, Man LK (2003). Abnormal peri-pubertal anthropometric measurements and growth pattern in adolescent idiopathic scoliosis: a study of 598 patients.. Spine (Phila Pa 1976).

[10] Nordwall A, Willner S (1975). A study of skeletal age and height in girls with idiopathic scoliosis..

[11] Grivas TB, Burwell RG, Mihas C, Vasiliadis ES, Triantafyllopoulos G (2009). Relatively lower body mass index is associated with an excess of severe truncal asymmetry in healthy adolescents: Do white adipose tissue, leptin, hypothalamus and sympathetic nervous system influence truncal growth asymmetry?. Scoliosis.

[12] Dardeno TA, Chou SH, Moon HS, Chamberland JP, Fiorenza CG (2010). Leptin in human physiology and therapeutics.. Front Neuroendocrinol.

[13] Hamrick MW, Ferrari SL (2008). Leptin and the sympathetic connection of fat to bone.. Osteoporos Int.

[14] Gautron L, Elmquist JK (2011). Sixteen years and counting: an update on leptin in energy balance.. J Clin Invest.

[15] Baldock PA, Allison S, McDonald MM, Sainsbury A, Enriquez RF (2006). Hypothalamic regulation of cortical bone mass: opposing activity of Y2 receptor and leptin pathways.. J Bone Miner Res.

[16] Wren AM, Small CJ, Abbott CR, Jethwa PH, Kennedy AR (2002). Hypothalamic actions of neuromedin U. Endocrinology.

[17] Hamrick MW, Della-Fera MA, Choi YH, Pennington C, Hartzell D (2005). Leptin treatment induces loss of bone marrow adipocytes and increases bone formation in leptin-deficient ob/ob mice.. J Bone Miner Res.

[18] Thomas T, Gori F, Khosla S, Jensen MD, Burguera B (1999). Leptin acts on human marrow stromal cells to enhance differentiation to osteoblasts and to inhibit differentiation to adipocytes.. Endocrinology.

[19] Qiu Y, Sun X, Qiu X, Li W, Zhu Z (2007). Decreased circulating leptin level and its association with body and bone mass in girls with adolescent idiopathic scoliosis.. Spine (Phila Pa 1976).

[20] Liu Z, Tam EM, Sun GQ, Lam TP, Zhu ZZ (2011). Abnormal Leptin Bioavailability in Adolescent Idiopathic Scoliosis - an Important New Finding.. Spine (Phila Pa.

[21] Morocz M, Czibula A, Grozer ZB, Szecsenyi A, Almos PZ (2011). Association study of BMP4, IL6, Leptin, MMP3, and MTNR1B gene promoter polymorphisms and adolescent idiopathic scoliosis.. Spine (Phila Pa 1976).

[22] Nduhirabandi F, du Toit EF, Lochner A (2012). Melatonin and the metabolic syndrome: a tool for effective therapy in obesity-associated abnormalities?.

[23] Burwell RG, Aujla RK, Grevitt MP, Dangerfield PH, Moulton A (2009). Pathogenesis of adolescent idiopathic scoliosis in girls - a double neuro-osseous theory involving disharmony between two nervous systems, somatic and autonomic expressed in the spine and trunk: possible dependency on sympathetic nervous system and hormones with implications for medical therapy.. Scoliosis.

[24] Akther A, Khan KH, Begum M, Parveen S, Kaiser MS (2009). Leptin: a mysterious hormone; its physiology and pathophysiology.. Mymensingh Med J.

[25] Janesick A, Blumberg B (2011). Endocrine disrupting chemicals and the developmental programming of adipogenesis and obesity.. Birth Defects Res C Embryo Today.

[26] van Harmelen V, Skurk T, Rohrig K, Lee YM, Halbleib M (2003). Effect of BMI and age on adipose tissue cellularity and differentiation capacity in women.. Int J Obes Relat Metab Disord.

[27] Janderova L, McNeil M, Murrell AN, Mynatt RL, Smith SR (2003). Human mesenchymal stem cells as an in vitro model for human adipogenesis.. Obes Res.

[28] Muruganandan S, Roman AA, Sinal CJ (2009). Adipocyte differentiation of bone marrow-derived mesenchymal stem cells: cross talk with the osteoblastogenic program.. Cell Mol Life Sci.

[29] Park WW, Suh KT, Kim JI, Kim SJ, Lee JS (2009). Decreased osteogenic differentiation of mesenchymal stem cells and reduced bone mineral density in patients with adolescent idiopathic scoliosis.. Eur Spine J.

[30] Cheng JC, Tang NL, Yeung HY, Miller N (2007). Genetic association of complex traits: using idiopathic scoliosis as an example.. Clin Orthop Relat Res.

[31] Gimble JM, Zvonic S, Floyd ZE, Kassem M, Nuttall ME (2006). Playing with bone and fat.. J Cell Biochem.

[32] Beresford JN, Bennett JH, Devlin C, Leboy PS, Owen ME (1992). Evidence for an inverse relationship between the differentiation of adipocytic and osteogenic cells in rat marrow stromal cell cultures.. J Cell Sci 102 (Pt.

[33] Abdallah BM, Kassem M (2011). New factors controlling the balance between osteoblastogenesis and adipogenesis..

[34] Zhang L, Su P, Xu C, Chen C, Liang A (2010). Melatonin inhibits adipogenesis and enhances osteogenesis of human mesenchymal stem cells by suppressing PPARgamma expression and enhancing Runx2 expression.. J Pineal Res.

[35] Bjorbak C, Lavery HJ, Bates SH, Olson RK, Davis SM (2000). SOCS3 mediates feedback inhibition of the leptin receptor via Tyr985.. J Biol Chem.

[36] Coen G (2004). Leptin and bone metabolism.. J Nephrol.

[37] Wlodarski K, Wlodarski P (2009). Leptin as a modulator of osteogenesis.. Ortop Traumatol Rehabil.

[38] Williams GA, Callon KE, Watson M, Costa JL, Ding Y (2011). Skeletal phenotype of the leptin receptor-deficient db/db mouse.. J Bone Miner Res.

[39] Bartell SM, Rayalam S, Ambati S, Gaddam DR, Hartzell DL (2011). Central (ICV) leptin injection increases bone formation, bone mineral density, muscle mass, serum IGF-1, and the expression of osteogenic genes in leptin-deficient ob/ob mice.. J Bone Miner Res.

[40] Iwaniec UT, Boghossian S, Lapke PD, Turner RT, Kalra SP (2007). Central leptin gene therapy corrects skeletal abnormalities in leptin-deficient ob/ob mice.. Peptides.

[41] Barrett JC, Fry B, Maller J, Daly MJ (2005). Haploview: analysis and visualization of LD and haplotype maps.. Bioinformatics.

[42] Hwang CS, Loftus TM, Mandrup S, Lane MD (1997). Adipocyte differentiation and leptin expression.. Annu Rev Cell Dev Biol.

