# Supplementary material for: Normal Leptin Expression, Lower Adipogenic Ability, Decreased Leptin Receptor and Hyposensitivity to Leptin in Adolescent Idiopathic Scoliosis
Source: PLoS One. 2012 May 15;7(5):e36648. doi: 10.1371/journal.pone.0036648 (PMC3352937; doi:10.1371/journal.pone.0036648)
Supplement: Table S1 — All primers for resequencing in genetic association study. (DOC) [file pone.0036648.s001.doc]

Table S1. All primers for resequencing.

| Coverage region | Primers for amplification | | Primers for sequencing | |
| --- | --- | --- | --- | --- |
|  | Forward | Reverse |  |  |
| exon1 | CTACCAGCCACCCCCAAAT | CACAGCCCAGCAGCAAATC | CTACCAGCCACCCCCAAAT | |
| exon2 | AGAGCACATTTCACAACAC | AATTAGAACCACTAACCCC | GATGGTAGCCAGAGCAGAA | |
| exon3 | GGGAACAGACTCCACTAAA  CATAAGACCCTAAGCCTCC  TTTGTGTGGTGGGTTCTTT  CAGGTGGGAAATGGTATG | CAAGAGGGGACAAGACAAC  CACTGCCATGTAATAAACC  TCAGCCTGATTAGGTGGTT  TCTCTCTGGCTCTTGGTG | GGAAAAGCAGGAATCTCGG | GCTTCAGGCTACTCCACAGA |
|  |  |  | CAGTTTCCAATCCCATAGA | AAAGAACCCACCACACAAA |
|  |  |  | AAGGGTAAAGAAGTTTGATA | AGAAGCCTGTTTTGTTGGA |
|  |  |  | AGTGTTCCTATTTGGGGCT | GTGAAACCCCGTCTCTACT |
|  |  |  | GGAATCTCGCTCTGTCATC | TTTCCCAGTCCTCTCAGCG |
