# Supplementary material for: Normal Leptin Expression, Lower Adipogenic Ability, Decreased Leptin Receptor and Hyposensitivity to Leptin in Adolescent Idiopathic Scoliosis
Source: PLoS One. 2012 May 15;7(5):e36648. doi: 10.1371/journal.pone.0036648 (PMC3352937; doi:10.1371/journal.pone.0036648)
Supplement: Table S2 — All primers and probes for each SNP in genetic association study. (DOC) [file pone.0036648.s002.doc]

Table S2. All primers and probes for each SNP.

| SNPs | amplification primers | | TaqMan probes | |
| --- | --- | --- | --- | --- |
|  | forward | reverse |  |  |
| L1 | GGGAACCCTGTGCGGATT | GCATACTCTCCTTACCGTGTGTGA | FAM-ATCTTTTCT***A***TGTCCAAGC-MGB | TET-ATCTTTTCT***G***TGTCCAAG -MGB |
| L2 | CACCCTCCCTCACCACTTTG | GGAATCTCGGAGACCAGCTTAG | FAM-CCTTGTATCCTG***C***ACCCAGGTGACT-TAMRA | TET-CCCTTGTATCCTG***T***ACCCAGGTGACTG-TAMRA |
| L3 | CTTCCCTTAACGTAGTCCTTGCA | CCTTCTCTAAGAGCTGCCACTTG | FAM-TGAGGTCCAG***C***TGCCACAGCAT-TAMRA | TET-TGAGGTCCAG***G***TGCCACAGCAT-TAMRA |
| L4 | AAATACCCCACCTTGGCACAT | GACTGGTGCTATAGGCTGGAGAA | FAM-ACCACTGGGAA***T***GCAAGATCCCTG-TAMRA | TET-ACCACTGGGAA***C***GCAAGATCCCT-TAMRA |
| L5 | TTTCTCTGCCTCTCGCTGTAACTC | CCTGTTTTGTTGGAAGGTTTGGT | FAM-CTGCTCA***C***ACTTTTA-MGB | TET-CTGCTCA***T***ACTTTTAC-MGB |
| L6 | CACAGCACCAGCCTCTTCTCTA | TAAAAGTGTGAGCAGTGAGTTACAGC | FAM-CCCTT***G***CCCTCCT-MGB | TET-CCCTT***T***CCCTCCTG-MGB |
