# Supplementary material for: Normal Leptin Expression, Lower Adipogenic Ability, Decreased Leptin Receptor and Hyposensitivity to Leptin in Adolescent Idiopathic Scoliosis
Source: PLoS One. 2012 May 15;7(5):e36648. doi: 10.1371/journal.pone.0036648 (PMC3352937; doi:10.1371/journal.pone.0036648)
Supplement: Table S3 — All primers for Real-time RT-PCR assay in cytological experiments. (DOC) [file pone.0036648.s003.doc]

Table S3. All primers for Real-time RT-PCR assay.

| Gene | Primers for amplification | |
| --- | --- | --- |
|  | Forward | Reverse |
| Leptin | ATGACACCAAAACCCTCATCAA | GAAGTCCAAACCGGTGACTTT |
| Leptin receptor | TGCCTTAGAGGATTATGGGTGTA | GTCATAGGTTGAATTTGGTGGC |
| SOCS3 | GGCCACTCTTCAGCATCTC | ATCGTACTGGTCCAGGAACTC |
| PPARγ2 | CCTATTGACCCAGAAAGCGATT | CATTACGGAGAGATCCACGGA |
| LPL | ACAAGAGAGAACCAGACTCCAA | AGGGTAGTTAAACTCCTCCTCC |
| APN | TATCCCCAACATGCCCATTCG | TGGTAGGCAAAGTAGTACAGCC |
| OCN | AGCAAAGGTGCAGCCTTTGT | GCGCCTGGGTCTCTTCACT |
| Runx2 | AGAAGGCACAGACAGAAGCTTGA | AGGAATGCGCCCTAAATCACT |
| ALP | AGCACTCCCACTTCATCTGGAA | GAGACCCAATAGGTAGTCCACATTG |
| OPN | ACACTCCTCGCCCTATTG | GATGTGGTCAGCCAACTC |
| GAPDH | AGAAAAACCTGCCAAATATGATGAC | TGGGTGTCGCTGTTGAAGTC |
